# Extracellular Vesicles-Mimetic Encapsulation Improves Oncolytic Viro-Immunotherapy in Tumors With Low Coxsackie and Adenovirus Receptor

**DOI:** 10.3389/fbioe.2020.574007

**Published:** 2020-09-16

**Authors:** Yonghui Zhang, Junyi Wu, Hailin Zhang, Jiwu Wei, Junhua Wu

**Affiliations:** ^1^Jiangsu Key Laboratory of Molecular Medicine, Medical School of Nanjing University, Nanjing, China; ^2^Henan Key Laboratory of Stem Cell Differentiation and Modification, Stem Cell Research Center, Henan Provincial People’s Hospital, People’s Hospital of Zhengzhou University, Zhengzhou, China; ^3^People’s Hospital of Henan University, Zhengzhou, China; ^4^Department of Hepatobiliary Surgery, Fujian Provincial Hospital, Fuzhou, China

**Keywords:** oncolytic virus, adenovirus, immune checkpoints, hepatocellular carcinoma, extracellular vesicles-mimetic

## Abstract

The oncolytic adenovirus (Adv) exhibited poor infection efficiency in tumor cells with low coxsackie and adenovirus receptor (CAR) on the cell surface, which limits the therapeutic efficacy of the Adv-mediated cancer gene therapy. In addition, the abundant adenovirus neutralizing antibodies also abrogate the viral infection of cancer cells. Therefore, novel strategies are required to overcome these two major hurdles to improve the Adv-mediated cancer virotherapy. We constructed a recombinant adenovirus expressing the extracellular domain of PD1 (Ad5-P). The 293T cells expressing VSV-G protein on the cell surface (293T-VSV-G) were infected with Ad5-P. Then Ad5-P infected 293T-VSV-G cells were harvested and squeezed stepwisely through a serial of polycarbonate membranes. Next, the extracellular vesicles-mimetic (EVM) encapsulated Ad5-P (EVM/VSV-G Ad5-P) were collected by density gradient centrifugation. In cell lines with low CAR expression, EVM/VSV-G Ad5-P showed a significantly improved infection efficiency, oncolytic ability, and soluble PD-1 production. In passively immunized mice with Ad5 neutralizing antibody, EVM/VSV-G Ad5-P successfully escaped from antibodies, and the soluble PD-1expression of Ad5-P was significantly prolonged. Finally, EVM/VSV-G Ad5-P treatment significantly improved the antitumor immune responses and prolonged survival of mice with HCC ascites. The EVM/VSV-G Ad5-P not only bypasses the limitation of low CAR expression in tumor cells to improve the viral entry, but also significantly protects the virus from the neutralization antibodies. The EVM encapsulation technology can be successfully used for loading of non-enveloped viruses to generate the extracellular vesicle-mimetic encapsulated viral particles. Our results provide a novel strategy in OVs manufacture to improve the efficacy of tumor oncolytic virotherapy.

## Introduction

In the last decade, remarkable achievements in tumor immunotherapy have been reported. Accumulated studies have confirmed that oncolytic viruses (OVs) can breakdown immune tolerance and shift “cold” tumors to “hot” tumors (Gujar et al., 2018). Oncolytic adenovirus is one commonly used vector for cancer therapy by locally expressing a gene of interest (Choi et al., 2011, 2013; Freytag et al., 2013). Adenovirus serotype 5 (Ad5) expressing immune checkpoint blockers, such as soluble PD-1, anti-PD-1, or anti-PD-L1, has been shown to strongly induce antitumor immune responses and significantly inhibit tumor growth, leading to prolonged survival of tumor-bearing mice (Shin et al., 2013; Tanoue et al., 2017; Kuryk et al., 2019; Zhang et al., 2019).

Although the application of oncolytic adenoviruses holds promise for cancer patients, some hurdles limit the therapeutic efficacy. Infection with Ad5 depends on the level of CAR expression on the cell surface, and previous studies have shown that CAR expression is downregulated during the growth of primary tumor cells, which limits Ad5 entry into tumor cells and thus its antitumor effect (Philipson et al., 1968; Miller et al., 1998; Li et al., 1999; Nigatu et al., 2013). In addition, neutralizing antibodies against Ad5 are present in more than 40% of adults (Nwanegbo et al., 2004), which may limit the application of Ad5. Moreover, adenovirus treatment elicits the production of neutralizing antibodies and triggers antiviral immunity, resulting in virus clearance, which limits the subsequent application of adenoviruses (Sumida et al., 2004). Therefore, strategies aiming to resolve these limitations will substantially increase the antitumor effect and applications of oncolytic adenoviruses.

To date, several methods have been developed to increase the Ad5 infection efficiency in cells with low CAR expression levels. One method involves covalent modification of the Ad5 capsid with artificial polymers, including polyethylene glycol (PEG) (Croyle et al., 2002; Cheng et al., 2003), polylactic glycolic acid (PLGA) (Matthews et al., 1999), polyethyleneimine (PEI) (Lee et al., 2014) and lipids (Lee et al., 2000; Wonganan and Croyle, 2010). In another method, Ad5 genes are modified to achieve retargeting, i.e., Ad5 with the insertion of Arg-Gly-Asp (RGD) peptide into the HI loop of the Ad5 fiber knob domain (Martínez-Vélez et al., 2019) or with the insertion of a chimeric Ad5/Ad35 fiber protein (Gall et al., 1996; Schroers et al., 2004). Although these methods increase the infection efficiency, the challenge of reducing antibody-mediated elimination still must be addressed.

A recent study showed that the exosome-associated adeno-associated virus (AAV) is resistant to AAV neutralizing antibodies (Gyorgy et al., 2014). In addition, the introduction of a targeting peptide on the exosome surface led to AAV retargeting (Lunavat et al., 2016; Martin and Raphael, 2017; Meliani et al., 2017; Schiller et al., 2018). Similarly, extracellular vesicles encapsulated oncolytic adenovirus (Adv) significantly increased the transduction ratio, and the infectious titer of the virus (Ran et al., 2016; Garofalo et al., 2018a, b, 2019). However, the yield of natural exosome-associated AAV or extracellular vesicles encapsulated Adv is relatively low, which limits its application prospects to a certain extent. In recent years, artificial exosome-mimetic (EM) or extracellular vesicles-mimetic (EVM) nanovesicle drug loading technology has been used to replace natural exosome technologies. The EM or EVM encapsulation technology is that in the extruder device, the cells carrying drugs are squeezed step wisely through a serial of polycarbonate membranes with a bore diameter at 10, 5, and 1 μm, and finally exosome or extracellular vesicle mimetic nanovesicles (EMs or EVMs) were formed. By this way, the (EMs or EVMs) are easily produced with relatively high yield, in the meanwhile, the therapeutic drugs are loaded into these nanovesicles. This novel EM technology overcomes the low yield of natural exosomes and generates EM constructs with various targets tropisms by using different cell lines (Jang et al., 2013; Hwang et al., 2015; Wu et al., 2018). The technique of EM or EVM is primarily used to encapsulate drugs (Tao et al., 2018; Wu et al., 2018; Garofalo et al., 2019), We wonder if the EVM strategy is also suitable for encapsulation of oncolytic viruses (OVs).

In this study, we generated a 293T cell line expressing a vesicular stomatitis Indiana virus G protein (VSV-G), a VSV membrane protein with a high affinity to most cell lines via low-density protein (LDL) receptor (Finkelshtein et al., 2013). Then, we employed the EVM technology to encapsulate oncolytic adenovirus expressing the extracellular domain of PD-1 (Ad5-P) in 293T-VSV-G and obtained EVM/VSV-G Ad5-P. We evaluated the virus particle yields, oncolytic efficiency, soluble PD-1 production, and infection capacity in the presence of Ad5 neutralizing antibodies. Finally, we investigated the immune activation and antitumor therapeutic efficacy of the EVM/VSV-G Ad5-P in an ascitic HCC mouse model.

## Materials and Methods

### Cell Lines and Cell Culture

The human hepatocellular carcinoma cell line HCC-LM3 and mouse hepatocellular carcinoma cell line H22 were obtained from the China Center for Type Culture Collection (Shanghai, China), authenticated by short tandem repeat (STR) analysis, and tested for mycoplasma contamination. The mouse hepatocellular carcinoma cell line Hepa1-6, human embryonic kidney cell line 293T, human T-cell leukemia cell line Jurkat, human chronic myeloid leukemia cell line K562, human alveolar adenocarcinoma cell line A549, mouse melanoma cell line B16-F10 and colon carcinoma cell line CT26.WT were obtained from the American Type Culture Collection (Manassas, VA, United States). 293T-VSV-G, an engineered 293T cell line that expresses VSV-G under control of the cytomegalovirus (CMV) early enhancer/chicken beta-actin (CAG) promoter, was engineered by our team by transducing cells with a lentiviral vector. Jurkat K562 and H22 cells were cultured in Roswell Park Memorial Institute 1640 (RPMI 1640) medium and other cells were cultured in Dulbecco’s Modified Eagle’s Medium (DMEM) supplemented with 10% fetal bovine serum, 2 mM L-glutamine, 100 units/ml penicillin, and 0.1 mg/ml streptomycin (all from Thermo Fisher Scientific, Gibco, Grand Island, NY, United States). All cells were maintained in a humidified incubator with an atmosphere containing 5% CO_2_ at 37°C.

### Construction of Recombinant Adenoviruses

The non-replicative adenovirus vector Ad5 and the oncolytic adenovirus Ad5-P were constructed using the ViraPower^TM^ Adenoviral Expression System and Gateway^®^ pENTR^TM^ Vectors (all from Life Technologies, Grand Island, NY, United States) as described previously (Zhang et al., 2019). Briefly, the obtained gene fragments were cloned into the adenovirus shuttle plasmid pENTR^TM^ using TOPO cloning technology. The cloned shuttle plasmid and the adenovirus backbone pAd/PL-DEST were combined to obtain the recombinant adenovirus vectors, pAd5-GFP and pAd5-P. For rescue of the recombinant viruses, the recombinant adenovirus vectors pAd5 and pAd5-P were linearized using the PacI restriction endonuclease (New England Biolabs, Ipswich, MA, United States), purified, and used to transfect 293 T cells using Lipofectamine 2000. Cells were continuously cultured for 10–14 days with complete culture medium containing 5% FBS until 70–80% of the cells exhibited cytopathy. The cells were collected and subjected to three freeze-thaw cycles. Then, the supernatant was harvested by centrifugation at 4,000 × g for 20 min and stored at −80°C as the virus seed stock. The 293T-VSV-G cells were used to amplify the non-replicative Ad5-GFP and the replicative Ad5-P. Cells were infected with viruses at an MOI of 5 for 72 h and then collected and subjected to repeated freeze-thaw cycles. Iodixanol density gradient centrifugation was performed to purify the virus.

Viral titers (MOI) were determined in 293T cells by serial dilution in 96-well plates for 4 days. Cells were observed under a fluorescence microscope. Wells with green fluorescent cells were defined as positive. The virus titer was calculated according to the following formula:

$$\text{TCID50}=10^{2+\left(S/N-0.5\right)}/\text{ml},$$

PFUs/ml=0.7×TCID50/ml,

where S is the total number of fluorescence-positive wells, and N is the number of replicates (Nicklin and Baker, 1999).

### Preparation of the Extracellular Vesicles-Mimetic Adenoviruses and VSV-G-Lenti-GFP

For the preparation of EVM adenoviruses, we first constructed 293T cells that expressed VSV-G (293T-VSV-G). Then, the 293T-VSV-G cells were inoculated onto 6, 10-cm^2^ culture dishes (1 × 10^7^ cells/plate). After overnight culture, the cells were inoculated with Ad5-GFP or Ad5-P viruses at 5 × 10^7^ PFUs and continuously cultured for 72 h. Next, the cells were collected, and the cell culture supernatant was removed. The cells were resuspended in 30 ml of DMEM, and the cell suspension was sequentially filtered through 10, 5, and 1 μm polycarbonate membranes (Whatman) using a mini-extruder (Avanti Polar Lipids). The filtered virus suspension was collected, placed in centrifuge tubes containing 15, 20, and 40% iodixanol cushions, and centrifuged at 100,000 × g for 90 min. Virus bands (EVM/VSV-G Ad5-GFP and EVM/VSV-G Ad5-P) located between 15 and 20% and between 20 and 40% iodixanol were collected. The virus suspension was dialyzed twice against a dialysis solution containing 5% glycerin, 1 mM MgCl_2_, 150 mM NaCl, and 10 mM Tris-HCl (pH 7.6). The virus suspension was aliquoted and stored in a −80°C freezer. Take out a tube of virus from the −80°C freezer and determine the viral titer. VSV-G-Lenti-GFP replication-defective lentiviral vectors were produced as previously described (Dull et al., 1998). Briefly, 293T cells were seeded in a 10-cm^2^ dish and transfected using Lipofectamine 2000 (Invitrogen/Thermo Fisher Scientific, Waltham, MA) according to the manufacturer’s instructions. Five micrograms of GFP transgene plasmid was cotransfected with 3 μg of pCMV-VSV-G (Vesicular stomatitis Indiana virus’s G protein expression plasmid), 5 μg of pCMV-Rev (Rev expression plasmid), and 5 μg of pMDLg/p.RRE (Gag/Pol expression plasmid) in 9 μl of Lipofectamine 2000 per dish. Supernatants were collected 24 and 48 h after transfection, cleared by low-speed centrifugation (4000 × g 10 min) and filtered through 0.45-μm-pore-size PVDF filters.

### Size Distribution Analysis of EVM/VSV-G Ad5-GFP

EVM/VSV-G Ad5-GFP were diluted with PBS which were prepared at a concentration of 20 μg/ml and the size of EVM/VSV-G Ad5-GFP was measured by NanoSight NS300 (Malvern Panalytical).

### Detection of the Virus Infection Efficiency

Cells were first inoculated in 24-well plates at a density of 1 × 10^5^ cells/well to determine the infection efficiency of the virus. Specific amounts of the corresponding non-replicative adenoviruses were added and cultured with the cells for 48 h at 37°C in a 5% CO_2_ atmosphere. Cells were observed and photographed under a fluorescence microscope. Cells were collected, and the ratio of GFP-positive cells was analyzed using flow cytometry. The ratio of GFP-positive cells among the 293T cells infected with Ad5-GFP or Ad5-P viruses was set to 1 to calculate the infection efficiency of viruses in other cells.

### Transmission Electron Microscopy (TEM) Imaging

EVM/VSV-G Ad5 and Ad5 viral solutions were placed on carbon-coated copper TEM grids (400 mesh, Agar Scientific) through dip-coating. Samples were dried at room temperature before observation. Samples were also stained with phosphotungstic acid. TEM observations were performed using a JEM-200CX microscope (JEOL, Japan).

### Neutralization Test

The adenovirus neutralizing antibody in adenovirus-immunized rabbit serum was provided by Shibing Wang (Zhejiang Sci-Tech University). The VSV-G neutralizing antibody was obtained from VSV immunized Mouse serum. Ascites samples obtained from animal experiments were processed and centrifuged at 400 × g and 12,000 × g for 10 min to remove cells and cell debris. The serum and ascites samples used in the neutralization test were inactivated at 56°C for 30 min. After 293T cells were inoculated in 24-well plates (1 × 10^5^ cells/well) and cultured at 37°C for 4 h, 100 μl of DMEM containing 1 × 10^5^ PFU viruses and the neutralizing serum or ascites samples were incubated at 37°C for 30 min. The virus-antibody mixture was added to 24-well plates that had been pre-inoculated with 293T cells (1 × 10^5^ cells/well) cultured at 37°C for 1 h. The viral supernatant was removed, and complete culture medium was added. After 48 h, fluorescence was observed, and the percentage of GFP-positive cells was analyzed using flow cytometry. The percentage of GFP-positive cells in the non-antibody treatment group was set to 100% to calculate the percentage of antibody neutralization in all treatment groups.

### Analysis of Cell Viability Using MTT Assays

The cell lines to be tested were inoculated in 96-well plates at a density of 10,000 cells/well and cultured at 37°C for 4 h. Specific amounts of the corresponding viruses were added. The viral suspension was removed after 4 h and replaced with complete culture medium. Cell culture was continued for another 72 h. Diluted MTT solution (100 μl, 1 mg/ml) was added to each well, and the culture was continued for 4 h. The supernatant was removed, 150 μl of isopropanol was added, and the samples were vortexed for 15 min. The absorbance was recorded at 570 nm. The absorbance of culture wells with non-treated cells was set to 100% cell viability to calculate the cell viability in other treatment groups.

### Quantitative PCR

The replicative Ad5-P or encapsulated EVM/VSV-G Ad5-P viruses were inoculated into Hepa1-6, B16/F10, CT26.WT, and H22 cells at an MOI of 5, and cells were collected at 6, 24, 36, 48, 60, or 72 h. Total DNA was extracted using a genome extraction reagent kit (TIANGEN Biotech). PowerUp^TM^ SYBR^TM^ Green Master Mix was used to amplify the DNA, quantified shuttle-Ad5-P plasmid was used as the standard, and Q-E1A F (5′-CCTTCTAACACACCTCCTGAGATACA-3′) and Q-EIA R (5′-CAGGCTCGTTAAGCAAGTCCTC-3′) were used as primers to detect the genome copies of viruses at different time points using Q-PCR. The virus copy number at 6 h in all treatment groups was set to 1 to calculate the fold increase in the virus copy number at different time points.

### Western Blotting

The protein concentration in the Ad5-GFP and EVM/VSV-G Ad5-GFP virus suspensions was measured after purification via density gradient centrifugation using the BCA method, and 80 μl of the suspension was mixed with 20 μl of 5× sample buffer (containing β-mercaptoethanol) (Beyotime, Hangzhou). Samples were incubated at 100°C for 5 min. Samples were separated on 12% SDS-PAGE gels and electrotransferred onto a polyvinylidene fluoride membrane (#03010040001; Roche). The membrane was blocked with TBS containing 10% non-fat milk for 30 min and then probed with a primary anti-VSV-G tag antibody (1:2,000, ab50549 Abcam, Cambridge, MA, United States) anti-CD63 antibody (1:1,000, ab59479 Abcam, Cambridge, MA, United States) anti-CD9 (1:1,000, 13403 Cell Signaling Technology, Danvers, MA, United States) and then a secondary horseradish peroxidase-labeled rabbit anti-mouse monoclonal antibody (1:5,000, A00160 GenScript Biotech Corp; Nanjing, China). The membrane was exposed to chemiluminescence reagent (WBKLS0500; Millipore, Billerica, MA, United States), and images were captured using a chemiluminescence imaging apparatus (ChampChemi 610; Sage Creation Science, Beijing, China).

### ELISA

A polystyrene microplate was pre-coated with His tag antibody (GenScript Biotech Corp., Nanjing, China) to quantify the levels of soluble proteins. One hundred microliters of supernatant or ascites was added to the plate and incubated for 2 h at 37°C. After washing, anti-PD-1 antibody (Sino Biological Inc., Beijing, China) and HRP-conjugated streptavidin were added and incubated for another 2 h at 37°C. 3,3′,5,5′-Tetramethylbenzidine (TMB) was used as the substrate, and the absorbance at 450 nm was recorded.

The concentration of interferon-gamma (IFN-γ) in ascites or plasma was quantified using a mouse IFN-γ ELISA kit according to the manufacturer’s instructions (BD Biosciences, Franklin Lakes, NJ, United States).

### Animal Experiments

For the HCC ascites model, specific-pathogen-free (SPF)-grade 8-week-old C57BL/6 male mice were purchased from Nanjing University Model Animal Institute. Next, 5 × 10^6^ H22 cells were intraperitoneally (i.p.) injected into the mice to construct an ascitic tumor model. On days 3, 9, 15, and 21, the mice were i.p. injected with recombinant adenovirus (5 × 10^8^ PFUs/mouse) or saline (as the control). Ascites samples were collected on days 11, 15, and 18, and the subsequent analysis was performed immediately. Mice that had been cured were rechallenged with 5 × 106 H22 cells on day 80, and naïve mice were used as the negative control. The mice were not treated after the adenovirus injection, and the survival of each mouse was recorded.

All animal experimental procedures were approved by the Animal Care Committee of Nanjing University in accordance with Institutional Animal Care and Use Committee guidelines.

### Flow Cytometry Analysis

Ascites fluid was collected at predefined time points, and cells were obtained by centrifugation. After washing, the harvested cells were stained with the following antibodies: anti-CD3-APC, anti-CD8a-PerCP-Cy^TM^5.5, anti-CD4-FITC, anti-NK1.1-FITC, and anti-CD274-PE (BD Biosciences, Franklin Lakes, NJ, United States). Cell lines were stained with PE-conjugated anti-CAR antibodies (Sino Biological Inc., Beijing, China). Then, the fluorescence intensity of cells was detected using a FACSCalibur flow cytometer (BD Biosciences, San Jose, CA, United States).

### IFN-γ ELISpot Assay

Peritoneal cells were harvested from murine ascites at the indicated time points. The number of IFN-γ-expressing cells was quantified using a Mouse IFN-γ ELISpot PLUS kit (3321-2AW-Plus; Mabtech, Nacka Strand, Sweden) according to the manufacturer’s instructions. Briefly, 2 × 105 cells were incubated in each well of a 96-well plate that had been pre-coated with anti-IFN-γ antibody for 20 h at 37°C. Unbound cells were removed by washing the plate, and biotinylated secondary antibody was added. The captured IFN-γ was visualized by adding HRP-conjugated streptavidin and the TMB substrate. An ELISpot reader (Autoimmun Diagnostika GmbH, Strassberg, Germany) was used to count the positive cells in each well.

### Statistical Analysis

Differences between samples indicated in the figures were tested for statistical significance by the Student *t*-test and *P* < 0.05 was considered statistically significant.

## Results

### The Infection Efficiency of Ad5 Is Dependent on CAR Expression in Different Cell Lines

**FIGURE 1:**
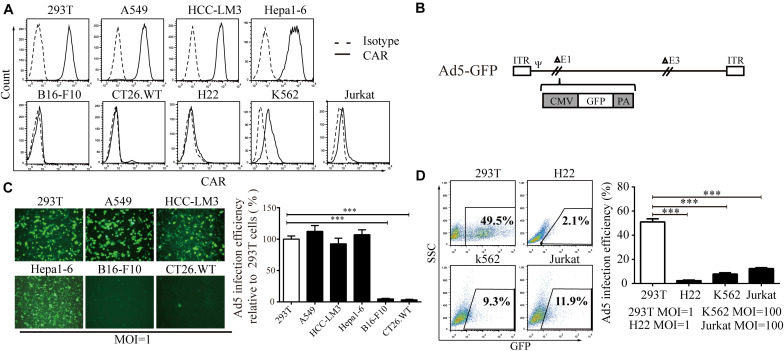
The relationship between CAR expression level and the Ad5 infection efficiency. **(A)** A series of cell lines (293T, A549, HCC-LM3, Hepa1-6, B16-F10, CT26.WT, H22, K562, and Jurkat cells) were stained with a monoclonal anti-CAR-PE antibody and subjected to flow cytometry to analyze the CAR expression level. A homologous IgG-PE antibody was used as the isotype control. **(B)** Genomic diagram of the non-replicative Ad5-GFP adenovirus. **(C)** 293T, A549, HCC-LM3, Hepa1-6, B16-F10, and CT26.WT cells were infected with Ad5-GFP for 72 h, and then, the cells were monitored under a fluorescence microscope (representative images are shown in the left panel) or subjected to FACS analysis. The infection efficiency in 293T cells was set to 100% to calculate the infection efficiency of Ad5 in each cell line. **(D)** 293T, H22, K562, and Jurkat cells were infected with Ad5-GFP at the indicated MOI. After 72 h, the cells were harvested and subjected to flow cytometry. The data are shown as the means ± SD. ^∗∗∗^*P* < 0.001.

First, we screened CAR expression in a variety of cell lines. We found that CAR was expressed in 293T cells and the A549, HCC-LM3, and Hepa1-6 cancer cell lines at a high level and in K562 and Jurkat cells at a low level but was barely detectable in B16-F10, CT26.WT, and H22 cells (Figure 1A). Using a non-replicative adenovirus expressing green fluorescent protein (Ad5-GFP, Figure 1B), GFP expression was observed in 50–60% of 293T, A549, HCC-LM3, and Hepa1-6 cells after Ad5-GFP infection. However, GFP expression was less than 5% in B16-F10 and CT26.WT cells after Ad5-GFP infection (Figure 1C). Consistently, in cell lines with low CAR expression, even when the multiplicity of infection (MOI) was increased 100-fold (MOI = 100), only 8.26 ± 0.64% and 12.08 ± 0.81% of K562 and Jurkat cells expressed GFP, respectively, significantly lower than the 49.5% in 293T cells infected with AD5-GFP at an MOI of 1 (Figure 1D). These results suggest that cells with low CAR expression limit the entry of Ad5.

### Preparation of Extracellular Vesicles-Mimetic EVM/VSV-G Ad5

**FIGURE 2:**
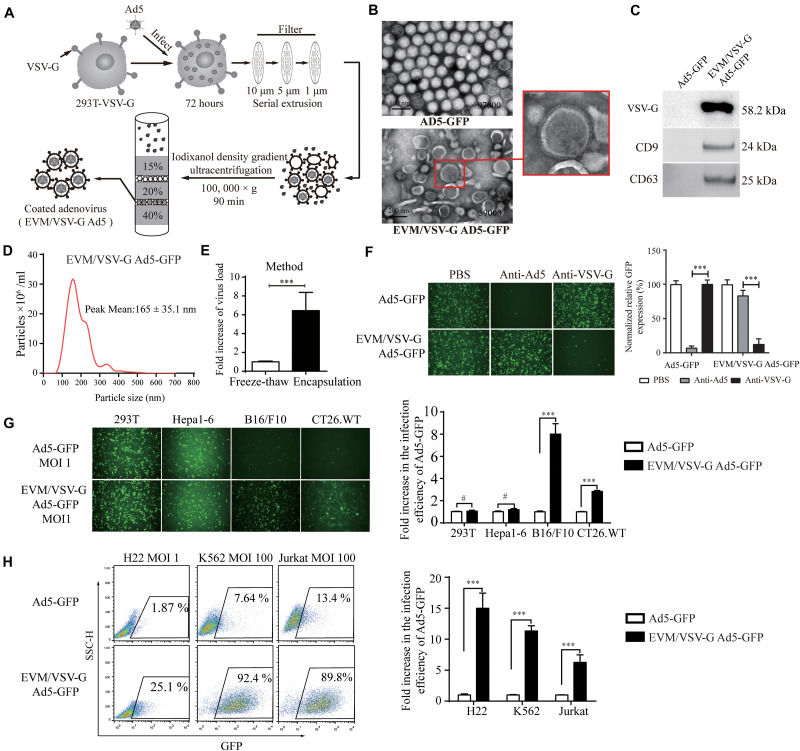
Preparationof extracellular vesicles-mimetic Ad5 (EVM/VSV-G Ad5). **(A)** Schematic diagram of EVM/VSV-G Ad5 preparation. **(B)** TEM photographs of Ad5-GFP and EVM/VSV-G Ad5-GFP viruses. The arrow in the lower panel points to the membrane protein spike of the particle. **(C)** Ad5-GFP and EVM/VSV-G Ad5-GFP viruses were harvested and purified by density gradient centrifugation, and then, CD63 CD9 and VSV-G protein was determined by western blotting. **(D)** Size distribution analysis of EVM/VSV-G Ad5-GFP by dynamic light scattering (NanoSight NS300). **(E)** 293T-VSV-G cells were infected with Ad5-GFP for 48 h, and then, the cells were evenly divided and subjected to either freeze-thaw cycles (open bar) or extracellular vesicles-mimetic production (filled bar). The viral titers were measured to calculate the amount of virus recovered. Fold increase compared with the freeze-thaw method is shown. **(F)** Ad5-GFP or EVM/VSV-G Ad5-GFP were incubated with Ad5 neutralizing antibodies or the VSV-G neutralizing serum then added to 293T cells for another 48 h. Then, green fluorescence-positive cells were either monitored with a fluorescence microscope (left panel) or analyzed by FACS (right panel). **(G)** 293T, Hepa1-6, B16/F10 and CT26.WT cells were infected with Ad5-GFP or EVM/VSV-G Ad5-GFP viruses at an MOI of 1, and GFP-positive cells were either monitored under a fluorescence microscope (upper panel) or analyzed by flow cytometry (lower panel). **(H)** H22, Jurkat and K562 cells were infected with Ad5-GFP or EVM/VSV-G Ad5-GFP at an MOI of 1 or 100 for 72 h. Then, GFP-positive cells were analyzed by FACS. Representative images of fluorescent cells from three independent experiments are shown (left panel). The results of the statistical analysis of the flow cytometry data are shown as the means ± SD (right panel). ^#^Not significant and ^∗∗∗^*P* < 0.001.

To overcome the limited entry in low-CAR cells, we sought to encapsulate the Ad5 viral particles into vesicle mimetics, we propagated EVM Ad5 in 293T cells expressing VSV-G (293T-VSV-G, Supplementary Figure S1), a ligand of LDL receptor commonly expressed by most tumor cells. The procedure is illustrated in Figure 2A and described in section “Materials and Methods.” The non-replicative adenoviruses expressing GFP protein (Ad5-GFP) were encapsulated in EVM/VSV-G, and the particles were analyzed by transmission electron microscopy (TEM). The size of naked Ad5-GFP viruses ranged from 70 and 90 nm, and the diameter of the EVM/VSV-G Ad5-GFP viral particles ranged from 100 and 200 nm, similar to extracellular vesicles (Figure 2B). We further confirmed that CD63, CD9, and VSV-G was only detected in EVM/VSV-G Ad5-GFP particles but not in the naked Ad5-GFP virus (Figure 2C). Dynamic light scattering analysis highlights size distribution and the peak value of 165 ± 35.1 nm for EVM/VSV-G Ad5 (Figure 2D). Finally, we determined the infective capability of EVM/VSV-G Ad5-GFP. Compared with the traditional freeze-thaw method, the infectious particle yield of the Ad5-GFP was increased to 6.4 ± 1.93 multiples by the EVM encapsulation (Figure 2E, the absolute yields are shown in Supplementary Figure S2.). Thus, we successfully generated the EVM Ad5 carrying VSV-G, CD63, and CD9.

### EVM/VSV-G Ad5-GFP Retains Viral Infection Capability in the Presence of Anti-Ad5 Antibody and Redirects Virus Entry via VSV-G

To determine if viral particles can be protected by EVM from neutralizing antibodies, the infectious efficiency of EVM/VSV-G Ad5-GFP was monitored in the presence of the anti-Ad5 antibody. Pre-incubation with the antibody did not abrogate the infectiousness of EVM/VSV-G Ad5-GFP, and the infection efficiency remained 83.67 ± 3.37%. However, the infection efficiency of the no encapsulation Ad5-GFP was massively reduced to 5.66 ± 2.1% (Figure 2F). These data suggest that EVM encapsulation protects the virus from neutralizing antibodies. While the virus was encapsulated by EVM, we postulated that the viral fiber was also enveloped in EVM. Therefore, the cell entry of EVM/VSV-G Ad5 may be switched from CAR to LDL-R via the binding of VSV-G anchored on EVM. To clarify the viral entry path, EVM/VSV-G Ad5-GFP was pre-incubated with anti-VSV-G antibody, the infection efficiency was markedly reduced to 12.16 ± 3.38%, while the anti-VSV-G antibody had little impact on the entry of naked Ad5-GFP, and the infection efficiency remained 97.66 ± 4.97% (Figure 2F). These results suggest that the infection of EVM/VSV-G encapsulated Ad5 is redirected by VSV-G.

### EVM/VSV-G Ad5-GFP Retains Infection Capability in CAR-Low Cell Lines

To exclude the impacts of viral replication and spread, we further generated non-replicative EVM/VSV-G Ad5-GFP to investigate the entry efficiency in cells with low CAR. We found that the infection capability of EVM/VSV-G Ad5-GFP was retained in B16/F10, CT26.WT, H22, K562, and Jurkat cells, However, in cell lines express low-level CAR, the infection efficiency of EVM/VSV-G Ad5-GFP was markedly higher than the naked Ad5-GFP, i.e., 8.00 ± 0.54-fold, 2.83 ± 0.04-fold, 14.99 ± 1.09-fold, 11.35 ± 0.37-fold, and 6.25 ± 0.58-fold higher, respectively (Figures 2G,H and Supplementary Figure S3A). Furthermore, in cells with high CAR expression, EVM/VSV-G Ad5-GFP exhibited an infection efficiency similar to Ad5-GFP (Figure 2G). Thus, the EVM/VSV-G encapsulation strategy sophistically bypasses the limited entry in cells lack of CAR.

### EVM/VSV-G Ad5-P Exhibits Higher Infection Efficiency, Improved Oncolysis, and Increased Soluble PD-1 Production in CAR-Low Cell Lines

**FIGURE 3:**
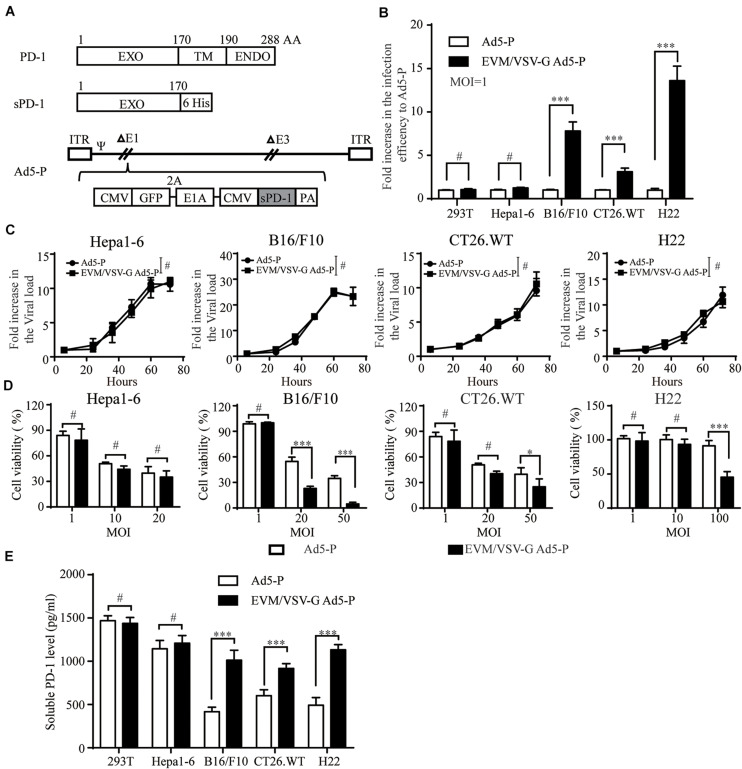
Effect of EVM/VSV-G encapsulation on infection efficiency, oncolysis, and soluble PD-1 production of Ad5-P. **(A)** A schematic diagram of the recombinant adenovirus Ad5-P. EXO, PD-1 extracellular domain; TM, PD-1 transmembrane domain; ENDO, PD-1 intracellular domain; 6 His, sequence of 6 His tags; sPD-1, soluble PD-1 protein containing the extracellular domain of PD-1 and 6 His; Ad5-P, recombinant Ad5 expressing sPD-1; EVM/VSV-G Ad5-P, EVM/VSV-G encapsulated Ad5-P. **(B)** 293T, Hepa1-6, B16.F10, CT26.WT, and H22 cells were infected with Ad5-P or EVM/VSV-G Ad5-P for 48 h and then subjected to FACS analysis. The fold increase in GFP-positive cells was compared between Ad5-P and EVM/VSV-G Ad5-P. The data are shown as the mean ± SD. **(C)** Hepa1-6, B16/F10, CT26.WT, and H22 cells were infected with Ad5-P or EVM/VSV-G Ad5-P for 6, 24, 36, 48, 60, or 72 h. Then, the cells were harvested, and changes in the virus copy number were analyzed using Q-PCR. The virus copies of each at 6 h was set to 1 to weigh virus copy numbers at different time points. The curves obtained depict the virus amplification. **(D)** Hepa1-6, B16/F10, CT26.WT, and H22 cells were infected with Ad5-P or EVM/VSV-G Ad5-P at the indicated MOIs, and 72 h later, cell viability was determined with MTT assays. **(E)** 293T, Hepa1-6, B16/F10, CT26.WT, and H22 cells were infected with Ad5-P or EVM/VSV-G Ad5-P at an MOI of 1 for 72 h, and then, the supernatant was collected to detect the soluble PD-1 levels via ELISA. The data are shown as means ± SD. ^#^Not significant, ^∗^*P* < 0.05, and ^∗∗∗^*P* < 0.001.

Next, we generated a recombinant replicative adenovirus expressing the extracellular domain of PD1 protein (Ad5-P) as depicted (Figure 3A) and described previously. Again, in low-CAR-expressing tumor cell lines, the infective efficiency of EVM/VSV-G Ad5-P was significantly higher than that of the naked Ad5-P, with values up to 8.7 ± 0.62-fold, 2.948 ± 0.039-fold, and 14.596 ± 1.56-fold higher in B16/F10, CT26.WT, and H22 cells, respectively (Figure 3B and Supplementary Figure S3B). However, the multiplication rate of EVM/VSV-G Ad5-P and Ad5-P was similar, either in the high CAR-expressing cell line Hepa1-6 or low-CAR-expressing cell lines, including B16/F10, CT26.WT, and H22 (Figure 3C and Supplementary Figure S4). These results suggest the EVM/VSV-G encapsulation alters virus entry but not affects virus propagation. Consistently, the oncolysis was enhanced by EVM/VSV-G Ad5-P in CAR-low cell lines (Figure 3D). In addition, the soluble PD-1 secretion was significantly increased in CAR-low cells compared to those infected with naked Ad5-P (Figure 3E). Taken together, EVM/VSV-G encapsulation increases the infectivity of Ad5-P leading to enhanced oncolysis and prolonged production of soluble PD-1 in CAR-low cancer cell lines.

### EVM/VSV-G Ad5-P Significantly Prolongs the Survival of Mice and Induces Long-Term Antitumor Immune Surveillance

**FIGURE 4:**
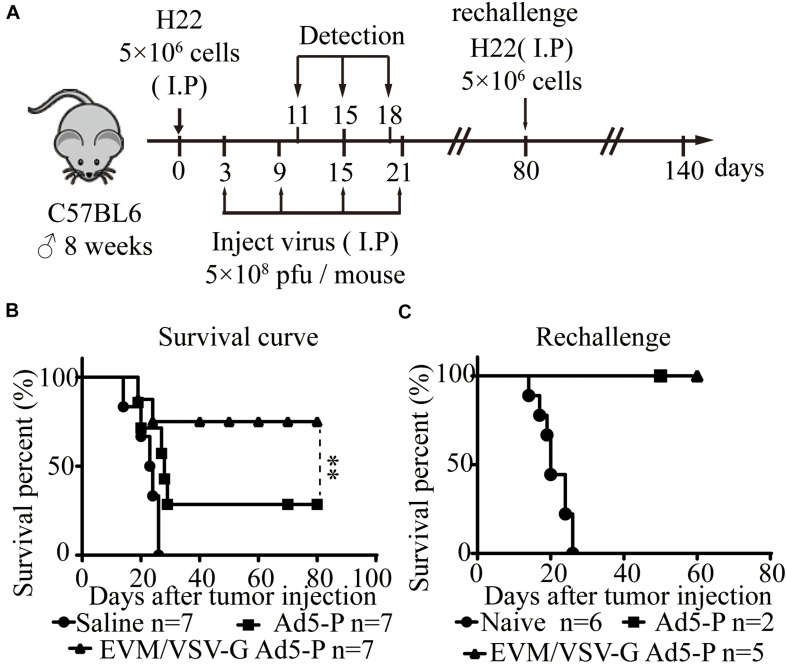
*In vivo* effect of EVM/VSV-G Ad5-P on the survival of mice and long-term antitumor immune surveillance. **(A)** A schematic diagram showing the *in vivo* experiment. **(B)** Eight-week-old C57BL6 male mice were randomly divided into three groups (*n* = 7 each group). On day 0, the mice received an intraperitoneal injection of 5 × 10^6^ H22 cells. On days 3, 9, 15, and 21, the mice received an intraperitoneal injection of 5 × 10^8^ PFU of either EVM/VSV-G Ad5-P (triangles) or Ad5-P (squares). The mice that received an intraperitoneal injection of an equal volume of PBS were used as controls (circles). **(C)** On day 80, the mice surviving after treatment with either Ad5-P (*n* = 2, squares) or EVM/VSV-G Ad5-P (*n* = 5, triangles) were rechallenged with 5 × 10^6^ H22 cells intraperitoneally, and the survival was monitored. Naïve mice (*n* = 6, circles) were used as controls. ^∗∗^*P* < 0.01.

Next, in an ascitic HCC mouse model, we investigated the therapeutic effect of EVM-VSV-G Ad5-P. The survival of mice treated with EVM-VSV-G Ad5-P was significantly prolonged, and five out of seven mice were cured (71.4%), while two out of the seven mice that received Ad5-P treatment were cured (28.5%) (Figure 4B). In addition, we performed a tumor rechallenge experiment in these cured mice. All the cured mice were resistant to the tumor rechallenge, whereas all the mice in the naive group died from tumor burden (Figure 4C). These results indicate that EVM/VSV-G Ad5-P induces long-term antitumor surveillance.

### EVM/VSV-G Ad5-P Significantly Improves the Antitumor Immune Response

**FIGURE 5:**
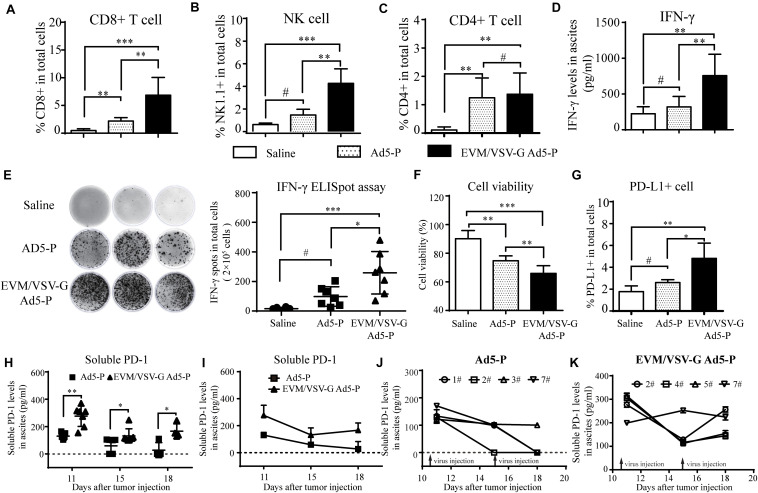
*In vivo* antitumor immune response of EVM/VSV-G Ad5-P. **(A–C)** Ascites fluid was obtained from the mice treated as described in Figure 4 on day 11, and the isolated ascites cells were incubated with CD3-APC together with either anti-CD8-PerCP-Cy^TM^5.5, anti-NK1.1-FITC, or anti-CD4-FITC antibodies. Then, the cells were subjected to FACS analysis. CD8+ or CD4+ T cells were identified as CD3/CD8 or CD3/CD4 double-positive cells. NK cells were CD3-APC-negative and NK1.1-positive cells. **(D)** The IFN-γ production levels in ascites on day 11 were measured via ELISA. The data are shown as means ± SD. **(E)** The IFN-γ-producing lymphocytes in ascites obtained on day 11 were detected with an ELISpot assay. The data shown are representative scanned photos of three mice from each group (left panel). The IFN-γ+ spots were counted and are shown as the means ± SD. **(F)** The viability of ascitic cells obtained on day 11 was determined by trypan blue staining. **(G)** Ascitic cells were labeled with PD-L1-PE, and the PD-L1-positive cells were analyzed by flow cytometry. The percentages of PD-L1-positive cells are shown. **(H)** The soluble PD-1 level in ascites fluid on day 11, 15, and 18 was detected via ELISA. The data are shown as means ± SD. h. ^#^Not significant, ^∗^*P* < 0.05, ^∗∗^*P* < 0.01, and ^∗∗∗^*P* < 0.001. **(I)** Ascites fluid was obtained from the mice, and the curves show the changes in the levels of soluble PD-1 in mouse ascites from the Ad5-P and EVM/VSV-G Ad5-P treatment groups. **(J)** Curves showing changes in the levels of soluble PD-1 in mouse ascites from the Ad5-P treatment group. **(K)** Curves showing changes in the levels of soluble PD-1 in mouse ascites from the EVM/VSV-G Ad5-P treatment groups.

Next, we investigated lymphocyte infiltration and the antitumor immune activation in ascites. We found that CD8+T and natural killer (NK) cells were significantly increased in ascites from the mice treated with EVM/VSV-G Ad5-P compared with that from mice treated with either saline or Ad5-P (Figures 5A,B). No difference in CD4+ T infiltration was observed between the two groups (Figure 5C). Consistently, EVM/VSV-G Ad5-P treatment resulted in high IFN-γ secretion and a high number of IFN-γ-producing cells in ascites (Figures 5D,E). Interestingly, EVM/VSV-G Ad5-P treatment not only induced PD-L1 expression in ascitic cells but also significantly increased the soluble PD1 production in ascites (Figures 5H,G). Moreover, we found that EVM/VSV-G Ad5-P exerted more cytotoxic efficacy against tumor cells (Figure 5F). These results indicate that EVM/VSV-G Ad5-P increases lymphocyte infiltration and enhances the antitumor immune responses.

### EVM/VSV-G Ad5-P Treatment Prolongs Production of Soluble PD-1 *in vivo*

**FIGURE 6:**
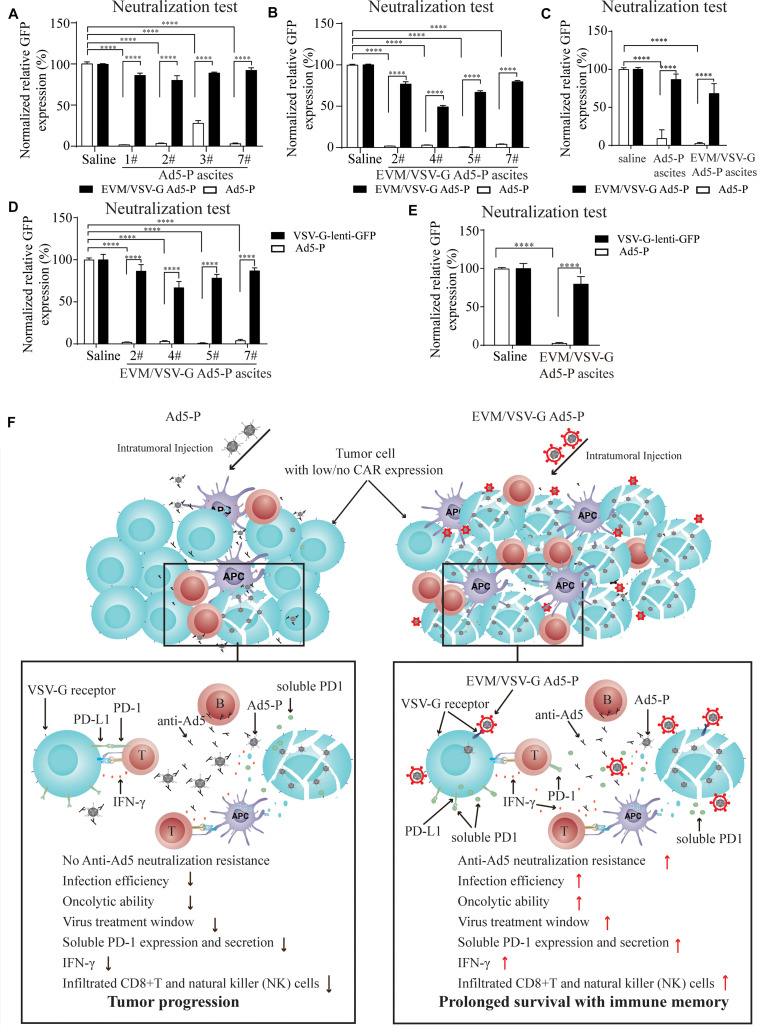
Effect of EVM/VSV-G encapsulation on the resistance to neutralizing antibodies and the secretion of soluble PD-1. **(A–C)** Ascites fluid from the mice treated with Ad5-P **(A)** or EVM/VSV-G Ad5-P **(B)** was harvested on day 15, and the supernatants were collected. Then, either Ad5-P or EVM/VSV-G Ad5-P viruses were incubated with the supernatant (filled bars) or PBS (open bars) for 30 min before being added to 293T cells. After 48 h, the number of GFP+ cells was determined by flow cytometry. The percentage of GFP positive cells was compared between cells infected with viruses incubated with PBS or supernatant. The mean and standard deviation of each group are shown **(C)**. **(D,E)** The supernatants of the ascites obtained from mice treated with EVM/VSV-G Ad5-P (*n* = 4) on day 15 were incubated with the lentivirus VSV-lenti-GFP for 30 min, and then, VSV-G-lenti-GFP viruses were added to 293T cells for 48 h. The percentages of GFP-positive cells were analyzed by flow cytometry **(D)**. The means and standard deviation in each group are shown **(E)**. ^****^P < 0.0001. **(F)** The underlying mechanism of EVM/VSV-G Ad5-P for *in vivo* immunotherapy. Compared with Ad5-P, the extracellular vesicles-mimetic encapsulated oncolytic adenovirus EVM/VSV-G Ad5-P has significantly enhanced the ability to resist anti-AD5 neutralization and promotes infection of low-CAR-expressing cancer cells, tumor oncolysis, PD-1 expression, and secretion, IFN-γ production, and CD8+T and NK cell infiltration. Therefore, the virus treatment window is prolonged, and a significantly high level of immune activation is triggered, eventually significantly prolonging the survival of mice and providing immune memory.

Since the expression of soluble PD-1 should be closely related to its antitumor effect of Ad5-P, we further analyzed the expression of soluble PD-1 in mouse ascites. We found that the soluble PD-1 concentrations in the Ad5-P group on days 18 and 15 were 27.59 ± 27.59 pg/ml and 58.68 ± 20.75 pg/ml, respectively, and both levels were significantly lower than the concentration of 130.2 ± 7.77 pg/ml measured on day 11 (Figure 5I). In the Ad5-P group, only one mouse had a soluble PD-1 concentration of 110.36 pg/ml on day 18, and the soluble PD-1 concentrations in ascites from other mice were lower than the detectable limit (Figure 5J). In the contrast, the soluble PD-1 concentrations in the EVM/VSV-G Ad5-P group on days 11, 15, and 18 were 276.4 ± 28.34 pg/ml, 132 ± 19.55 pg/ml, and 166.4 ± 26.66 pg/ml, respectively. Although the soluble PD-1 concentrations on days 15 and 18 were significantly decreased compared with that on day 11, the concentration measured on day 18 was increased compared with that on day 15, and soluble PD-1 was persistently secreted (Figure 5I). The curve in Figure 6K shows the changes in soluble PD-1 levels in each mouse in the EVM/VSV-G Ad5-P group over time. After the virus injection on day 15, the soluble PD-1 level in ascites from the EVM/VSV-G Ad5-P group was slightly decreased in one mouse, whereas the levels in the other three mice were significantly increased (Figure 5K). These results suggest that EVM/VSV-G Ad5-P significantly prolongs the viral expression of soluble PD-1 *in vivo* which may benefit the blockade of the checkpoint of PD-L1/PD-1.

### EVM/VSV-G Encapsulation Protects Ad5-P From Neutralizing Antibodies

Next, we wanted to know if EVM/VSV-G encapsulation protects Ad5 from neutralizing antibodies. To this end, ascites fluid from the mice treated with either Ad5-P or EVM/VSV-G Ad5-P was harvested. We found that ascites from mice in both groups significantly abrogated the infection with Ad5-P virus (Figures 6A–C), suggesting the Ad5-P or the EVM/VSV-G Ad5-P treatment induces the generation of anti-Ad5 antibodies, which neutralize Ad5-P. However, the infection capacity of EVM/VSV-G Ad5-P was retained (Figures 6A–C), indicating that EVM/VSV-G Ad5-P is resistant to neutralizing antibodies. To exclude the potential impact of the VSV-G neutralizing antibodies that might be induced by EVM/VSV-G Ad5-P, we compared the neutralizing efficacy of the ascites on Ad5-P and VSV-G-lenti-GFP virus. We found that the neutralizing antibodies abrogated Ad5 infection but could not prevent VSV-G-lenti-GFP virus infection (Figures 6D,E). Taken together, EVM/VSV-G encapsulation markedly protects Ad5-P from neutralizing antibodies, which may prolong the virus persistence *in vivo*.

## Discussion

Type V adenovirus is one of the most used vector for oncolytic virotherapy of cancer. However, the low expression of CAR, the specific infection receptor of Ad5, and the pre-existing neutralizing antibodies limit the therapeutic efficacy of Ad5. In this study, we present a novel method for oncolytic adenovirus preparation using EVM technology. Ad5-P encapsulated in EVM/VSV-G retained the infectivity in CAR-low cancer cells, and successfully escaped from being neutralized by anti-Ad5 antibodies. The two distinct advantages of EVM/VSV-G Ad5-P benefit persistent expression of PD-1, leading to markedly improved antitumor immune activation and prolonged the survival of mice with ascitic HCC (Figure 6F).

In the current study, the oncolytic virus is a type V adenovirus with E1B-55kDa and E3 deletion. Theoretically, p53 is a tumor suppressor gene and can induce cell cycle arrest and/or apoptosis in response to foreign DNA synthesis such as that during adenovirus infection. Given that the E1B-55kDa protein inactivates the tumor suppressor protein p53, the adenovirus with an E1B-55kDa deletion cannot inactivate p53 in normal cells and is therefore unable to replicate efficiently. However, in cancer cells with p53 mutation, the virus with E1B deletion may replicate efficiently and induce oncolysis.

Although the majority of normal epithelial tissues in humans express CAR, CAR expression might be downregulated or lost during malignancy progression, which substantially limits the entry of Ad5 oncolytic adenoviruses into tumor cells and hinders their antitumor effects (Douglas et al., 2001). To date, several strategies have been developed to increase the infection efficiency of Ad5 in low-CAR-expressing cells. For instance, the fiber proteins of adenovirus can be genetically modified to retarget other cell surface receptors, e.g., the chimeric viruses AdF5/35, Ad5/7 or Ad5-RGD (Gall et al., 1996; Martínez-Vélez et al., 2019). We found that EVM/VSV-G Ad5-P robustly increases the infectivity of adenoviruses in cells with low CAR expression. VSV-G protein mediates virus entry into all cell types that have been tested to date, and this protein has been used extensively in gene transduction and gene therapy (Finkelshtein et al., 2013). This technology successfully broadens the routes for viral entry into cells. In addition, the VSV-G protein can be replaced with other specific cancer-targeting proteins or polypeptides to target specific tumors.

The EVM/VSV-G Ad5 preparation technology is easy to manipulate. The cells infected with the virus were sequentially squeezed/filtered through a 10, 5, and 1 μm polycarbonate membranes. In this way, the yield of the infectious particles was significantly higher than with the conventional freeze-thaw method. Since the freeze-thaw method may not guarantee the complete release of viral particles from the host cells, a portion of the viruses likely attach to cell debris and thus are difficult to harvest via viral purification. However, using the EVM technique, we obtained and harvested the final 1 μm particles from the host cells; therefore, the yield of the infectious particles was increased. In addition, several freeze-thaw cycles may reduce virus viability, whereas the EVM technique avoids this issue. Thus, the EVM technique provides a great advantage for oncolytic adenovirus production. Whether this technique is suitable for other OVs, including enveloped or non-enveloped viruses, requires further investigation.

During viral therapy, the generation of neutralizing antibodies in the hosts not only prevents the spread of the OV within the tumor mass but also limits repeat application of the OVs. Previous studies have shown that artificial polymers, such as polyethylene glycol (PEG) (Cheng et al., 2003), polylactic glycolic acid (PLGA) (Matthews et al., 1999), polyethyleneimine (PEI) (Lee et al., 2014), or lipids (Lee et al., 2000), can be utilized to modify the adenovirus capsid protein by forming a complex that is resistant to neutralizing antibodies against Ad5. However, the introduction of polymers might result in safety concerns. In addition, polymers such as PEI bind to the cell membrane through electric charge and thus may lack tissue specificity. Several other studies have shown that exosome-encapsulated AAVs are resistant to AAV neutralizing antibodies (Gyorgy et al., 2014; Meliani et al., 2017). To date, very few extracellular vesicles-encapsulated Ad5 have been few described (Ran et al., 2016; Garofalo et al., 2018a, b, 2019). However, the low yield of the naturally occurring exosomal viral particles severely limits its application (Maguire et al., 2012; Meliani et al., 2017; Schiller et al., 2018). EVM encapsulated Ad5 was easier to produce and consists of one envelope layer encapsulating the Ad5 capsid, which is similar to the extracellular vesicles-encapsulated Ad5. Although anti-Ad5 neutralizing antibodies were generated by the viral treatment and severely blocked naked Ad5 infection, these antibodies did not affect EVM/VSV-G Ad5-P infection. Therefore, even when injected repeatedly, EVM/VSV-G Ad5-P effectively infected tumor cells and generated soluble PD-1 production to block the PD-L1/PD-1 checkpoint. This advantage is critical for OVs-based cancer therapy. Although the VSV-G protein is also an exogenous protein with potent immunogenicity presented in extracellular vesicles-mimetic, we found that few anti-VSV-G neutralizing antibodies were generated in mice. The reason may be that the amount of VSV-G carried by EVM/VSV-G Ad5-P was not enough to induce neutralizing antibodies against VSV-G. If the virus injections are increased, the titer of VSV-G neutralizing antibodies anticipatedly are increased. Noteworthily, the delayed production of sufficient amount of VSV-G neutralizing antibodies prolonged the therapeutic window of the oncolytic virus.

Theoretically, the extracellular vesicles-mimetic encapsulation technology can achieve retargeting of non-enveloped viruses to any receptor. In this study, VSV-G is used as a proof-of-principle to verify the feasibility of the EVM/OVs strategy. Therefore, our study supports the similar strategies to replace the VSV-G with “ligands” that have both tumor targeting and low immunogenicity.

Increasing evidences show that oncolytic virotherapy is mainly achieved by inducing an effective and specific anti-tumor immune response (Lichty et al., 2014). The generation of an effective anti-tumor immune response is tightly related to whether oncolytic viruses could force tumor cells to undergo immunogenic cell death (ICD) (Bommareddy et al., 2018). For example, adenovirus vector expressing soluble PD1 does not have antitumor effect when used alone, but the combination of this virus and ganciclovir, which can induce tumor cell death, induces an antitumor immune response (Shin et al., 2013). Although EVM encapsulation had no direct impact on viral propagation and spread, but it (with the same amount of virus) did increase the entry of viruses and subsequently enhanced oncolysis resulting in enhanced ICD-mediated antitumor immune responses. Furthermore, EVM encapsulation protected Ad5 from being neutralized by antibodies, which in turn prolonged the therapeutic window. In this way, more viral progenies along with the significantly prolonged expression of soluble PD1, further recruited more lymphocyte infiltration and sufficiently maintained the activity of tumor-specific T cells for a period of time. These features of extracellular vesicles-mimetic encapsulated oncolytic virus (EVM-OVs) not only improved the antitumor immune activity but also elicited long-term antitumor immune surveillance. Thus, the EVM technique can not only be used for OV-mediated immunotherapy, but also for manufacture of immune vaccines against viruses.

In addition to adenovirus, the EVM technique may also be suitable for producing other non-enveloped viruses. However, we would not exclude the possibility of EVM for the enveloped viruses, which requires further investigation. Given the distinct advantages of EVM encapsulation of OVs, it deserves further development of devices for large-scale production of EVM-OVs to meet the needs of clinical application soon.

The EVM encapsulation technology can be successfully employed in the preparation of OVs. The EVM-OVs show the potential to efficiently bypass the limitation of low-expression of virus infection receptors. Moreover, EVM-OVs retain the infectivity in the presence of virus neutralizing antibodies, prolong the therapeutic duration of OVs. These distinct advantages of EVM-OVs have practical implications for OV manufacture and future clinical applications.

## Data Availability Statement

All datasets generated for this study are included in the article/Supplementary Material.

## Ethics Statement

The animal study was reviewed and approved by the Animal Care Committee of Nanjing University.

## Author Contributions

JHW and JWW conceived the study, designed the experiments, and supervised the project. YZ, JYW, and HZ performed the experiments and analyzed the data. JHW, YZ, and JWW wrote the manuscript. All authors critically reviewed and approved the manuscript.

## Conflict of Interest

The authors declare that the research was conducted in the absence of any commercial or financial relationships that could be construed as a potential conflict of interest.
