# Supplementary material for: Extracellular Vesicles-Mimetic Encapsulation Improves Oncolytic Viro-Immunotherapy in Tumors With Low Coxsackie and Adenovirus Receptor
Source: Front Bioeng Biotechnol. 2020 Sep 16;8:574007. doi: 10.3389/fbioe.2020.574007 (PMC7525182; doi:10.3389/fbioe.2020.574007)
Supplement: Supplementary file 1 [file Data_Sheet_1.PDF]

Supplementary Material

1 Supplementary Figures

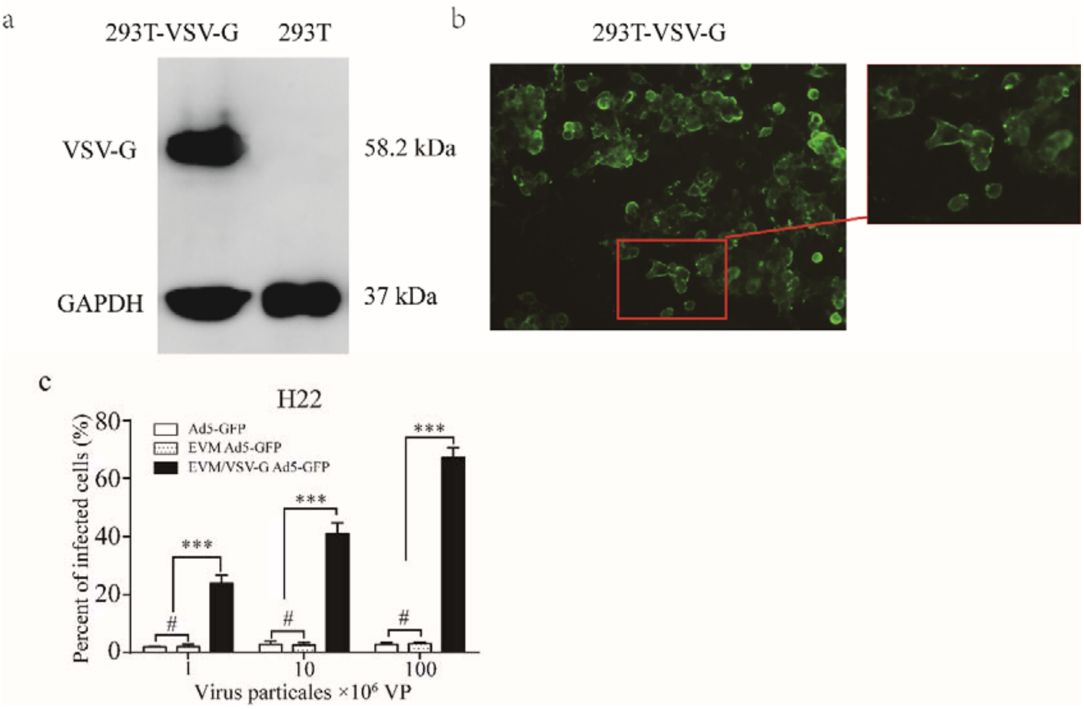

Supplementary Figure 1. The expression and localization of VSV-G in 293T-VSV-G Cell. a. 293T and 293T-VSV-G were harvested and total protein was extracted. Then the VSV-G protein was detected by western blot. b. Immunofluorescence image of 293T-VSV-G Cell. The VSV-G protein was detected with mouse anti-VSV-G at 293T-VSV-G Cells. The secondary antibody is goat anti-mouse IgG-FITC. c. H22 cells were infected with Ad5-GFP, EVM Ad5-GFP or EVM/VSV-G Ad5-GFP at 1×106 VP, 10×106 VP and 100×106 VP for 72 hours. Then, GFP-positive cells were analyzed by FACS. The results of statistical analysis of the flow cytometry data are shown as the means ± SD. # not significant, *** P < 0.001.

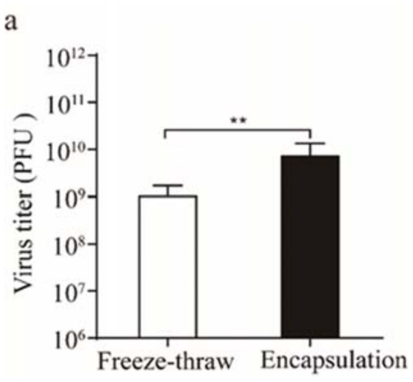

Supplementary Figure 2. Cell-specific yield of the EM encapsulated virus. 293T-VSV-G cells were infected with Ad5-GFP for 48 h, and then, the cells were evenly divided and subjected to either freeze-thaw cycles (open bar) or extracellular vesicles-mimetic production (filled bar). The viral titers were measured to calculate the amount of virus recovered.

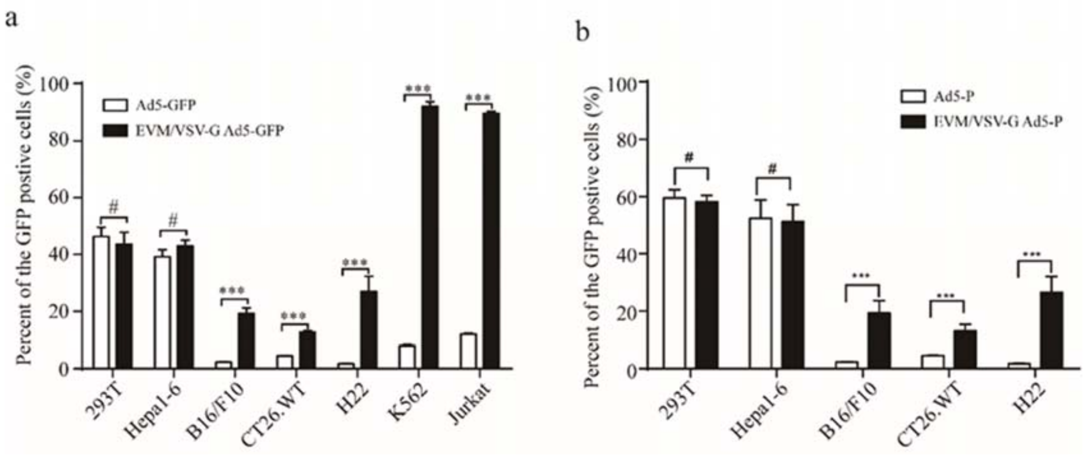

Supplementary Figure 3. Infection ability of EVM/VSV-G Ad5-GFP in CAR-low cell lines. a. 293T, Hepa1-6, B16/F10 and CT26.WT cells were infected with Ad5-GFP or EVM/VSV-G Ad5-GFP viruses at an MOI of 1, and GFP-positive cells were analyzed by flow cytometry. H22, Jurkat and K562 cells were infected with Ad5-GFP or EVM/VSV-G Ad5-GFP at an MOI of 1 or 100 for 72 h. Then, GFP-positive cells were analyzed by FACS. b. 293T, Hepa1-6, B16/F10 CT26.WT and H22 cells were infected with Ad5-P or EVM/VSV-G Ad5-P viruses at an MOI of 1, and GFP-positive cells were analyzed by flow cytometry. The results of statistical analysis of the flow cytometry data are shown as the means ± SD. # not significant, * $P < 0.05$, ** $P < 0.01$, and *** $P < 0.001$

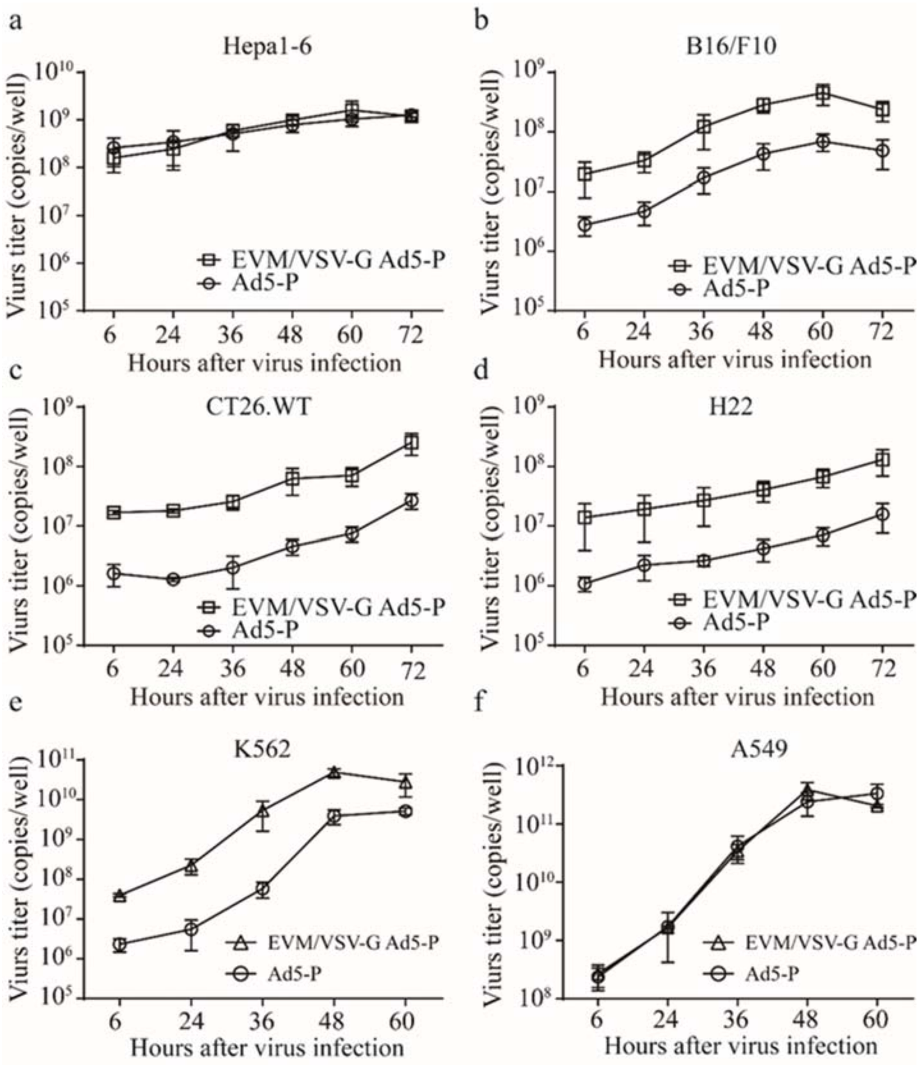

Supplementary Figure 4. Replication capacity of Ad5-P and EVM/VSV-G Ad5-P in various murine or human cell lines. Hepa1-6, B16/F10, CT26.WT, H22, K562 and A549 cells were infected with Ad5-P or EVM/VSV-G Ad5-P for 6, 24, 36, 48, 60, or 72 h. Then, the cells were harvested, and changes in the virus copy number were analyzed using Q-PCR. The curves obtained depict the virus amplification.

2. Supplementary Method

Preparation of 293T-VSV-G cell line

293T-VSV-G cell was engineered by our team by transducing cells with a lentiviral vector. Briefly, 293T cells were seeded in a 10 cm^2 dish and transfected using Lipofectamine 2000 (Invitrogen/Thermo Fisher Scientific, Waltham, MA) according to the manufacturer's instructions. Five micrograms of VSV-G transgene plasmid (Expressing lentiviral vector genome FigS5) was cotransfected with 3 µg of pCMV-VSV-G (Vesicular stomatitis Indiana virus's G protein expression plasmid), 5 µg of pCMV-Rev (Rev expression plasmid), and 5 µg of pMDLg/p.RRE (Gag/Pol expression plasmid) in 9 µl of Lipofectamine 2000 per dish. Supernatants were collected 24 and 48 h after transfection, cleared by low-speed centrifugation (4000 ×g 10 min), and filtered through 0.45-µm-pore-size PVDF filters. Store

at 4℃ and use with 2-3 days. Prepared two 10 cm2 dish of 293T cells before one days of virus vector infection. Removed the medium and wash one time with PBS. add 6 ml of virus to cell and incubate at 37℃ for 4 hours. Change normal medium incubate at 37℃ for 48 hours. Change the normal medium with 1.5ug/ml of puromycin incubate for 48 hours. Change the normal medium with 0.5ug/ml of puromycin for long time culture.

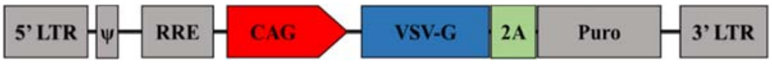

Figure S5 Schematic diagram of expressing VSV-G lentiviral vector genome. VSV-G was controlled by the cytomegalovirus early enhancer/chicken beta-actin promoter (CAG); Fusion expression of VSV-G and puromycin resistance gene is linked by the 2A Self-Cleaving Peptide.

3. Soluble PD1 sequence

Soluble PD1 Nucleotide sequence

ATGCAGATCCCACAGGCGCCCTGGCCAGTCGTCTGGGCGGTGCTACAACTGGGCTGGCG
GCCAGGATGGTTCTTAGACTCCCCAGACAGGCCCTGGAACCCCCCCACCTTCTCCCCAGC
CCTGCTCGTGGTGACCGAAGGGGACAACGCCACCTTCACCTGCAGCTTCTCCAACACAT
CGGAGAGCTTCGTGCTAAACTGGTACCGCATGAGCCCCAGCAACCAGACGGACAAGCTG
GCCGCCTTCCCCGAGGACCGCAGCCAGCCCGGCCAGGACTGCCGCTTCCGTGTCACACA
ACTGCCCAACGGGCGTGACTTCCACATGAGCGTGGTCAGGGCCCGGCGCAATGACAGCG
GCACCTACCTCTGTGGGGCCATCTCCCTGGCCCCCAAGGCGCAGATCAAAGAGAGCCTG
CGGGCAGAGCTCAGGGTGACAGAGAGAAGGGCAGAAGTGCCCACAGCCCACCCCAGCC
CCTCACCCAGGCCAGCCGGCCAGTTCCAAACCCTGGTGCACCACCACCACCACCACTAA

Soluble PD1 Amino acid sequence

MQIPQAPWPVVWAVLQLGWRPGW (PD1 Signal peptide)
FLDSPDRPWNPPTFSPALLVVTEGDNATFTCSFSNTSESFVLNWYRMSPSNQTDKLAAFPED
RSQPGQDCRFRVTQLPNGRDFHMSVVRARRNDSGTYLCGAISLAPKAQIKESLRAELRVTER
RAEVPTAHPSPSPRPAGQFQTLV (PD1 Extracellular domain)
HHHHHH. (His tag)
